# Aseptic Meningitis and Papillary Edema: Uncommon Presentation of Myelin Oligodendrocyte Glycoprotein Antibody-Associated Disease (MOGAD)

**DOI:** 10.7759/cureus.98547

**Published:** 2025-12-05

**Authors:** Naoko Kojima, Naoki Yamada, Ichiro Kuki, Hiroshi Sakuma, Shin Okazaki

**Affiliations:** 1 Department of Pediatric Neurology and Neurolinguistics, Osaka City General Hospital, Osaka, JPN; 2 Department of Brain and Neurosciences, Tokyo Metropolitan Institute of Medical Science, Tokyo, JPN

**Keywords:** aseptic meningitis, autoimmune encephalitis, cerebrospinal fluid, fever, headache, methylprednisolone, mogad, mog associated antibody disease (mogad), papillary edema, pleocytosis

## Abstract

Myelin oligodendrocyte glycoprotein antibody-associated disease (MOGAD) is an autoimmune disease characterized by central nervous system demyelination. We report a case of prolonged aseptic meningitis in which anti-MOG antibodies were subsequently detected. In addition, we review previously reported cases of MOGAD that initially presented with symptoms of meningitis. MOGAD with meningitis without neurological symptoms or parenchymal brain lesions was rare and characterized by high cerebrospinal fluid (CSF) opening pressures.

## Introduction

Myelin oligodendrocyte glycoprotein antibody-associated disease (MOGAD) is an autoimmune disease characterized by central nervous system demyelination. It presents a diverse clinical picture, including optic neuritis (ON), transverse myelitis, acute disseminated encephalomyelitis (ADEM), and cerebral cortical encephalitis. In pediatric cases, the most frequently observed phenotype is ADEM, characterized by encephalopathic manifestations including fever, seizures, headache, disturbances of consciousness, and behavioral abnormalities. MRI typically demonstrates multiple lesions involving both the white and gray matter. An infectious prodrome may precede the onset of ADEM manifestations [1]. On the other hand, aseptic meningitis has been increasingly reported as the initial presentation of MOGAD in children. In recent years, however, many aspects remain ambiguous [2]. We report a case of prolonged aseptic meningitis in which anti-MOG antibodies were subsequently detected. In addition, we review previously reported cases of MOGAD that initially presented with symptoms of meningitis.

## Case presentation

**Figure 1:**
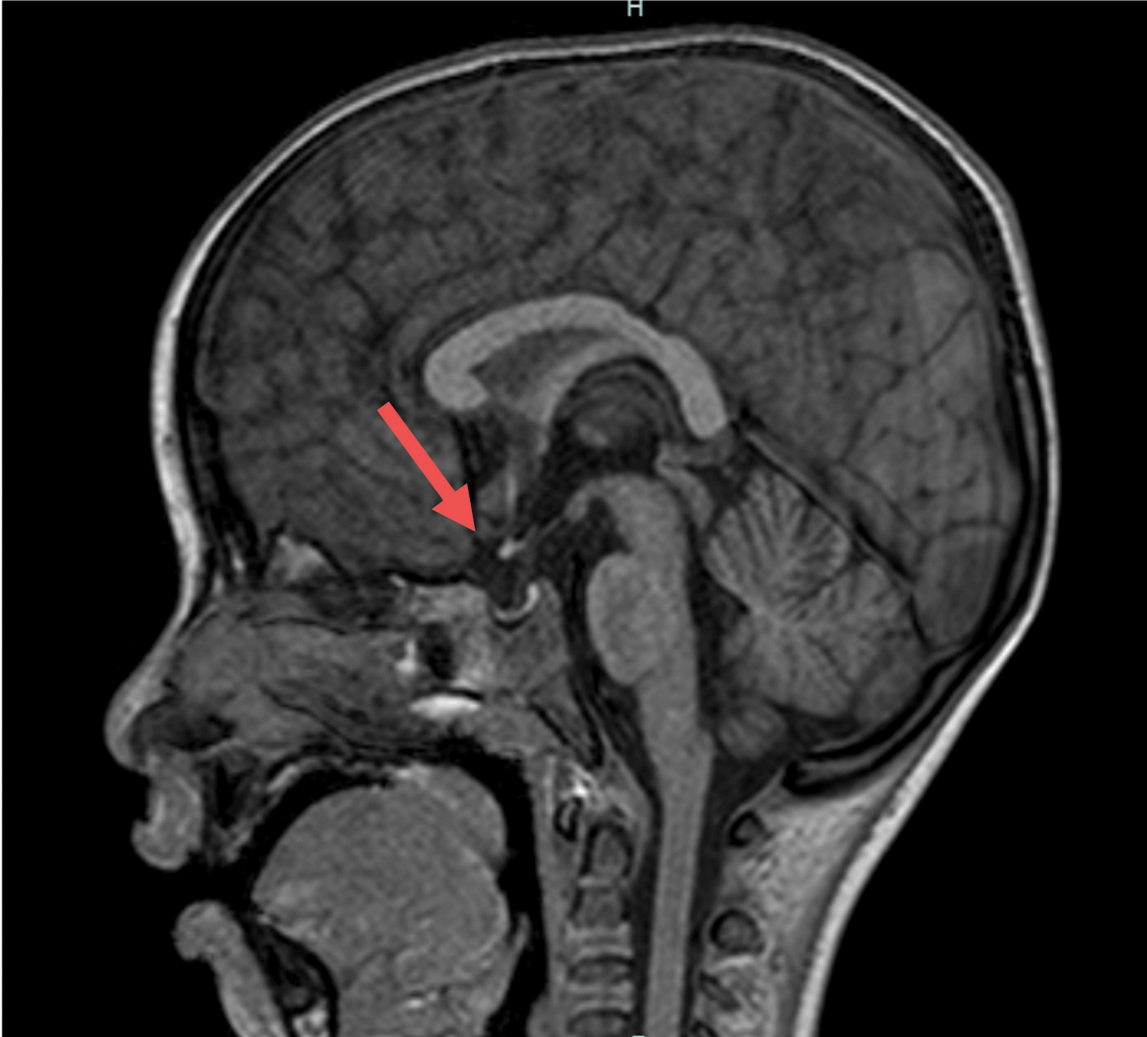
Pre-therapeutic magnetic resonance imaging of the brain Magnetic resonance imaging of the head showed empty sella. There were no findings of optic neuritis, transverse myelitis, acute disseminated encephalomyelitis, or cerebral cortical encephalitis.

**Figure 2:**
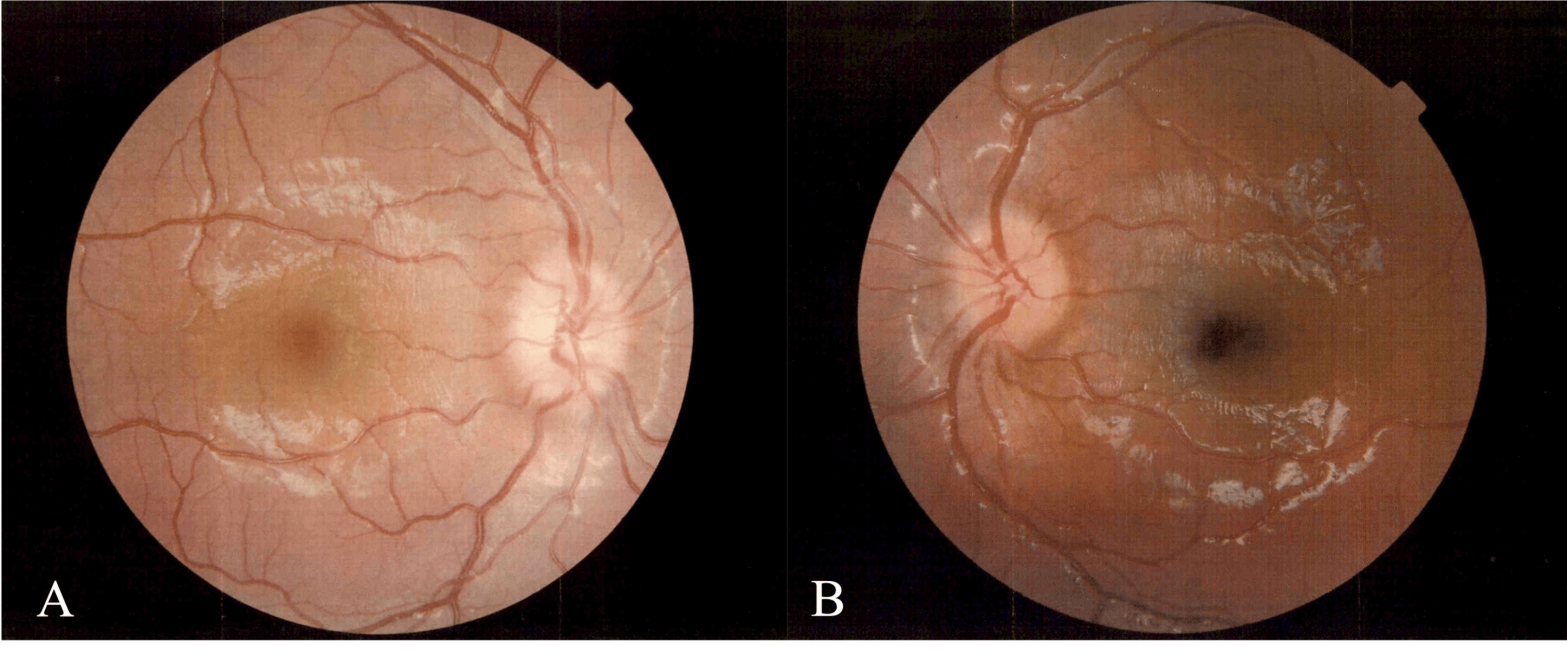
Ophthalmic fundus imaging Ophthalmological examination revealed bilateral papillary edema of the fundus (A: right, B: left).

**Figure 3:**
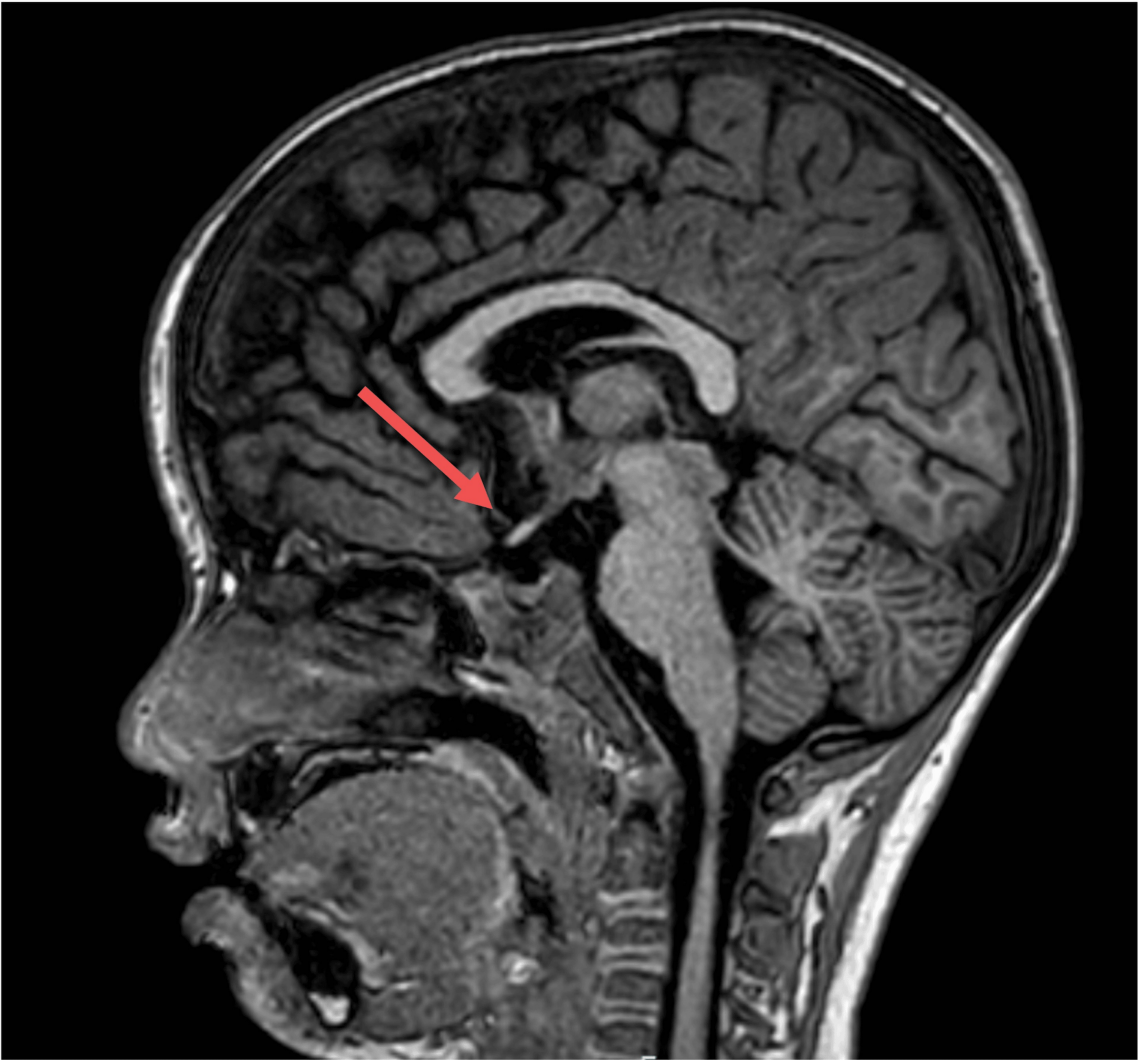
Post-therapeutic magnetic resonance imaging of the brain Magnetic resonance imaging of the head showed a reduction in the empty sella.

A previously healthy five-year-old boy with persistent vomiting and fever, experiencing headaches for 14 days prior to admission, presented to our hospital. Neurological examination revealed marked nuchal stiffness and no other abnormalities. Peripheral blood tests revealed leukocytosis (21.54 × 10³/μL) and a mild elevation of C-reactive protein (CRP), and a magnetic resonance imaging (MRI) of the head showed empty sella (Figure 1). No contrast agent was used in the MRI examination. Ophthalmological examination revealed bilateral papillary edema of the fundus (Figure 2), and a cerebrospinal fluid (CSF) analysis revealed leukocytosis (123/µL) and an elevated opening pressure of 32 cmH₂O. (Table 1 shows the laboratory results with corresponding reference ranges.) Bacterial culture of cerebrospinal fluid and multiplex PCR testing were performed. Multiplex polymerase chain reaction (PCR) is a test for 14 types of bacteria, viruses, and fungi that are major pathogens causing meningitis and encephalitis. The results of both the multiplex PCR and bacterial culture were all negative. Glycerol and acetazolamide were initiated, and ceftriaxone was administered as an antibiotic. Since neither bacteria nor viruses responsible for meningitis were detected in the cerebrospinal fluid or blood tests performed at admission, ceftriaxone was discontinued. Despite the treatment, the patient continued to have a fever and nuchal stiffness on the fifth day of admission. Furthermore, no improvements were observed in the CSF analysis, with an initial pressure of more than 35 cmH₂O and a CSF cell count of 116/µL. Oligoclonal bands were detected in the CSF. Subsequently, intravenous methylprednisolone (IVMP) therapy was initiated for meningitis with an autoimmune mechanism, and the symptoms dissipated the following day. After three courses of IVMP, the papillary edema resolved. The CSF opening pressure and cell count improved to 24 cmH₂O and 20/µl, respectively. Moreover, the head MRI scan showed a reduction in the empty sella (Figure 3). Hence, the patient was discharged on hospital day 23 without any neurological deficits. Anti-MOG antibodies in the serum and spinal fluid obtained on admission were positive. Ten weeks after discharge, the oligoclonal bands became negative, and the CSF opening pressure improved to 12.5 cmH₂O. No new neurological symptoms were observed at six months after discharge.

**Table 1:** Laboratory data The table summarizes the laboratory findings at the time of the patient's hospital admission.

Test	Result	Normal Range
Cerebrospinal Fluid (CSF)		
Opening pressure	32 mmHg	< 20 mmHg
Cell count	123 /μL	< 5 /μL
– Mononuclear cells (%)	34.10%	—
– Polymorphonuclear cells (%)	65.90%	—
Glucose	55 mg/dL	71–90 mg/dL
Protein	34 mg/dL	15–45 mg/dL
Neopterin	214.7 nmol/L	< 20 nmol/L
Anti-MOG antibody (CSF)	Positive	Negative
Blood		
White blood cell count (WBC)	20.3 × 10³/μL	4.0–10.0 × 10³/μL
C-reactive protein (CRP)	4.27 mg/dL	< 0.30 mg/dL
Anti-MOG antibody (Serum)	Positive	Negative

## Discussion

MOGAD with meningitis, without other neurological symptoms or parenchymal brain lesions, is rare. Table 2 summarizes previous reports [1-8] and our case of aseptic meningitis as the initial presentation of MOGAD. Among the reports, 13 of the 16 patients had fever, headache, and neck rigidity persisting for more than two weeks. Notably, papillary edema was observed in two patients, a finding that was also present in the current case. Seizures were present in five patients; seizures were observed in five patients; however, none of them developed other neurological symptoms. The present case was characterized by high CSF opening pressure. Although cases of increased intracranial pressure have been reported in MOGAD with phenotypes such as ADEM and ON [9], many cases of high CSF opening pressure have been found even in patients with aseptic meningitis alone. The mechanism of increased intracranial pressure is unknown, but inflammation of the meninges may increase the production of spinal fluid or impede its absorption. All patients received immunotherapy, except one who recovered spontaneously at an early stage, with a good therapeutic response. Eight of the cases listed in the table later developed neurological symptoms or imaging abnormalities. There was a trend towards progression to ADEM and ON in patients with a longer time before starting immunotherapy. In cases of meningitis with prolonged symptoms and high intracranial pressure, it is important to consider MOGAD and initiate immunotherapy promptly.

**Table 2:** Characteristic of aseptic meningitis as the initial presentation of MOGAD ADEM: acute disseminated encephalomyelitis; CSF: cerebrospinal fluid; F: female; IVIG: intravenous immunoglobulin; IVMP: intravenous immunoglobulin; M: male; MMF: mycophenolate mofetil; MOGAD: Myelin oligodendrocyte glycoprotein antibody-associated disease; ON: optic neuritis.

		Age/Gender	Fever	Headache	Vomiting	Seizure	Papillary oedema	CSF opening pressure (cm H2O)	CSF WBC (×10^6^/L)	The duration of symptoms (day)	Immune treatment			Other MOGAD after diagnosis		Relapse
This study		4/M	+	+	+		＋	32	123	15	IVMP			No		No
Lin et al. 2022 [1]		13/M	+	+	+			26	392	24	IVMP IVIG			ADEM ON		Yes
		5/M		+	+			30	44	13	IVMP IVIG			NA		No
Wong et al. 2022 [2]		4/F	+		+	+		>32	16	18	IVMP IVIG			ADEM		No
		12/F		+		+	+	60	147	30	IVMP			ADEM		No
Udani et al. 2021 [3]		2/F	+			+		NA	61	25	IVMP			ON		No
		5/M	+	+		+		NA	158	17	IVMP			ADEM		No
		4/F	+	+				NA	8	21	IVMP			ADEM		No
Song et al. 2022 [4]		11/M	+	+	+			NA	200	20	IVMP			ADEM		NA
		10/F	+	+				NA	488	21	IVMP			No		No
		3/M	+			+		NA	240	15	IVMP			No		No
Gu et al. 2023 [5]		13/M	+					15	52	11	IVMP IVIG			No		No
		12/M	+					15	40	24	IVMP IVIG AZA			No		No
		7/F	+					38.5	175	23	IVMP IVIG			No		No
Hino-Fukuyo et al. 2023 [6]		13/F	+	+				NA	406	9	None			No		No
Yousseff et al. 2023 [7]		12/M		+	+		+	35	75	21	IVMP			ON		No
Laura et al. 2024 [8]		8/M		+	+			75	63	21	IVMP IVIG			ON		Yes

## Conclusions

In the present case, we initially suspected aseptic meningitis of viral or other non-bacterial origin based on the clinical presentation and laboratory findings and initiated empirical treatment. However, due to the persistence of symptoms, an autoimmune etiology was considered, and immunotherapy was commenced, resulting in the rapid resolution of symptoms, with no progression to other manifestations. Previous reports suggest that delayed initiation of immunotherapy is associated with a higher likelihood of progression to other phenotypes within the MOGAD spectrum; however, further studies are needed to clarify this association. In cases presenting with symptoms of aseptic meningitis, the time to diagnosis tends to be prolonged. In patients with persistent meningeal symptoms, it is important to consider MOGAD and initiate immunotherapy promptly.
